# First Molecular Detection of Porcine Cytomegalovirus (PCMV) and Porcine Lymphotropic Herpesvirus (PLHV) in Domestic Pigs in Poland

**DOI:** 10.3390/pathogens14040396

**Published:** 2025-04-18

**Authors:** Piotr Cybulski, Wojciech Socha, Artur Jabłoński, Radosław Kondratiuk, Weronika Rybkowska, Tomasz Stadejek, Magdalena Larska

**Affiliations:** 1 Goodvalley Agro S.A., Dworcowa 25, 77-320 Przechlewo, Poland; radoslaw.kondratiuk@goodvalley.com; 2 Department of Virology and Viral Animal Diseases, National Veterinary Research Institute, Partyzantów 57, 24-100 Puławy, Poland; wojciech.socha@piwet.pulawy.pl (W.S.); m.larska@piwet.pulawy.pl (M.L.); 3 Department of Pathology and Veterinary Diagnostics, Institute of Veterinary Medicine, Warsaw University of Life Sciences—SGGW, Nowoursynowska 159C, 02-776 Warsaw, Poland; artur_jablonski@sggw.edu.pl (A.J.); weronika_gorecka@sggw.edu.pl (W.R.); tomasz_stadejek@sggw.edu.pl (T.S.)

**Keywords:** swine, herpesviruses, PCMV, PLHV-1, Poland

## Abstract

Contrary to extensively studied porcine alphaherpesvirus (SuHV-1/PRV), betaherpesvirus (SuHV-2/PCMV) and Gammaherpesvirinae (SuHV-3/PLHV-1, SuHV-4/PLHV-2, SuHV-5/PLHV-3) infections remain unexplored in the swine population in Poland. The aim of this study was to characterise the prevalence of infections and local strains from each major herpesvirus subfamily on a large-scale weaner farm located in Poland. Nasal swabs collected from pigs at 6, 8 and 10 weeks of age were tested for the presence of herpesvirus infections using nested PCR specific to the pan-herpesvirus DNA polymerase (DPol) gene. The amplicons obtained from the positive samples were sequenced using the Sanger method. In total, 60% of the pigs were positive for herpesviruses, including 35.6% for porcine cytomegalovirus (SuHV-2/PCMV) and 24.4% for porcine lymphotropic herpesvirus type 1 (SuHV-3/PLHV-1). The infection rate was lowest in 6-week-old pigs—20% (6.7%—PCMV, 13.3%—PLHV-1) in comparison to 8-week-old—80% (53.3%—PCMV, 26.7% PLHV-1)—and 10-week-old pigs—80% (46.7%—PCMV, 33.3%—PLHV-1). No correlation between PCMV and PLHV-1 infections and coinfections with IAV, PRV1 or PRRSV was observed. Sequence analysis of both PLHV-1 and PCMV showed high genetic uniformity. Additionally, PLHV-1 isolates showed a close relationship to strains isolated from wild boar in Poland and pigs in Germany in recent years. In summary, our study confirmed the presence of both PLHV-1 and PCMV infections occurring early in piglet development, probably after passive immunity cessation.

## 1. Introduction

**Figure 1:**
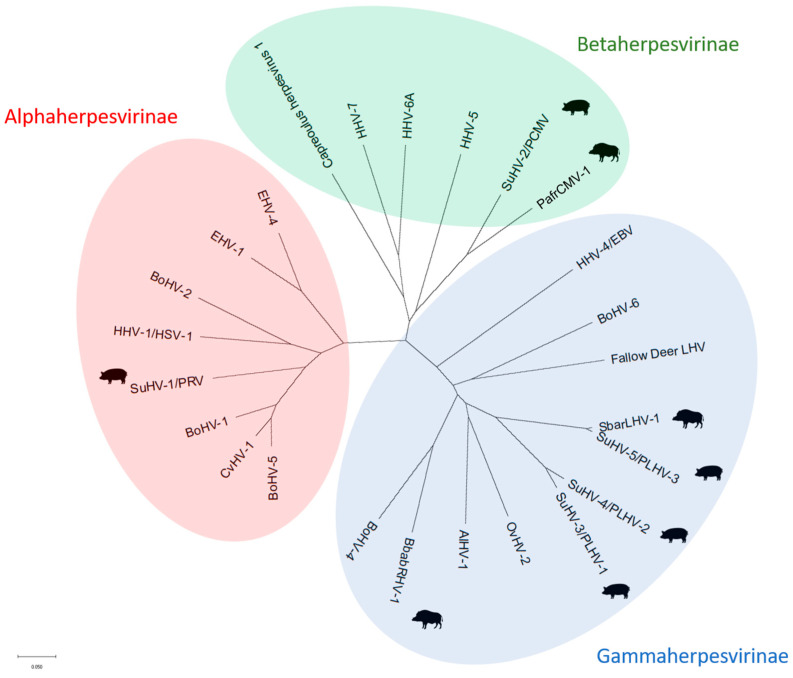
Neighbour-joining phylogenetic tree of selected herpesviruses of humans and animals constructed based on partial sequencing of DNA polymerase gene. Viruses infecting mainly swine or wild suids are marked with respective silhouettes. AlHV-1—alcelaphine (wildebeest) herpesvirus 1, BbabRHV-1—*Babyrousa babyrussa* rhadinovirus 1, BoHV-1—bovine alphaherpesvirus 1, BoHV-2—bovine alphaherpesvirus 2, BoHV-4—bovine gammaherpesvirus 4, BoHV-5—bovine alphaherpesvirus 5, BoHV-6—bovine gammaherpesvirus 6, CvHV-1—cervid herpesvirus 1, EHV-1—equid alphaherpesvirus 1, EHV-4—equid alphaherpesvirus 4, fallow deer LHV—fallow deer lymphotropic gammaherpesvirus, HHV-1/HSV-1—human alphaherpesvirus 1/herpes simplex virus 1, HHV-4/EBV—human gammaherpesvirus 4/Epstein-Barr virus, HHV-5—human betaherpesvirus 5, HHV-6A—human betaherpesvirus 6A, HHV-7—human betaherpesvirus 7, OvHV-2—ovine herpesvirus 2, PafrCMV-1—*Phacochoerus africanus* (warthog) cytomegalovirus 1, SbarLHV-1—*Sus barbatus* (bearded pig) lymphotropic herpesvirus 1, SuHV-1/PRV—Suid herpesvirus 1/pseudorabies virus, SuHV-2/PCMV—Suid herpesvirus 2/porcine cytomegalovirus, SuHV-3/PLHV-1—Suid herpesvirus 3/porcine lymphotropic herpesvirus 1, SuHV-4/PLHV-2—Suid herpesvirus 4/porcine lymphotropic herpesvirus 2, SuHV-5/PLHV-3—Suid herpesvirus 5/porcine lymphotropic herpesvirus 3.

Comprising more than 100 members recognised thus far, *Herpesviridae* is a family of enveloped, double-stranded linear DNA, icosahedral viruses that affect a broad range of animal species and humans. All of the family members are characterised by the capacity for developing viral latency with periodic reactivation associated with the influence of several stress factors. Based on sequence analysis and the unique biological properties of herpesviruses, the International Committee on Taxonomy of Viruses (ICTV) divided the family into three subfamilies: *Alphaherpesvirinae*, *Betaherpesvirinae*, and *Gammaherpesvirinae*, with at least one member relevant to domestic pigs (*Sus (S.) scrofa domestica*) in each cluster [1] (Figure 1).

*Suid alphaherpesvirus 1* (SuHV-1; virus name: pseudorabies virus—PRV, formerly Aujeszky’s disease virus—ADV) is a member of the subfamily *Alphaherpesvirinae*, genus *Varicellovirus,* which is characterised by rapid lytic viral replication and latency in sensory ganglia. *Suid betaherpesvirus 2* (SuHV-2; virus name: porcine cytomegalovirus—PCMV) belongs to the subfamily *Betaherpesvirinae*, genus *Roselovirus*. The virus establishes latency in non-neural tissues, and its relatively slow replication cycle causes infected cell enlargement. The latter subfamily of *Herpesviridae*, *Gammaherpesvirinae*, consisting of so-far characterised *Suid gammaherpesvirus 3* (SuHV-3; virus name: porcine lymphotropic herpesvirus 1—PLHV-1), *Suid gammaherpesvirus 4* (SuHV-4; virus name: porcine lymphotropic herpesvirus 2—PLHV-2) and *Suid gammaherpesvirus 5* (SuHV-5; virus name: porcine lymphotropic herpesvirus 3—PLHV-3), all belonging to the genus *Macavirus*, establish their latent infection in porcine lymphoid organs and lymphocytes.

The clinical relevance of PRV as a causative agent of Aujeszky’s disease (AD) has been thoroughly studied to date, including the development of highly efficacious marker vaccines and differential ELISA tests collectively allowing successful eradication programmes based on discrimination between vaccinated and infected animals [2,3,4,5]. Contrary to the amassed comprehensive data concerning AD, knowledge on the epidemiology, pathogenesis, and immunity of other porcine herpesviruses still remains severely limited.

PCMV has been detected on commercial farms in most swine-producing countries [6,7,8,9,10,11]. The virus is transmitted via the oronasal route and causes inclusion body rhinitis (IBR) [12,13,14], with clinical signs usually observed in about 4-week-old piglets; however, congenital transmission has also been demonstrated [14,15]. Despite its high prevalence, PCMV infection in pigs is seldom recognised as the sole contagious factor causing substantial financial losses in modern swine production. The detection of the virus has also been confirmed in wild boar (*S. scrofa*) [16]; nevertheless, its epidemiology remains largely unknown. Although natural infections with herpesviruses are considered to be highly host-specific [1,17], a growing body of research employing non-human primates as a model has demonstrated substantially reduced survival of PCMV-infected xenotransplants and a considerable risk of xenoinfectivity [18,19,20,21].

PLHV-1 and PLHV-2 were discovered in the spleen and leukocytes collected from clinically healthy pigs reared on commercial farms located in Germany and Spain in the late 1990s [22]. PLHV-3 was identified in the early 2000s during an investigation carried out on the blood and spleen samples obtained from healthy German pigs [23]. Even though the viruses have been reported at a relatively high prevalence in domestic pigs worldwide [24,25,26,27,28,29], peer-reviewed works demonstrating the mode of transmission are lacking in scientific literature. Similarly, despite academic research on the prevalence of the PLHVs in feral members of the genus *Sus* [30,31,32], solid evidence on the epidemiology of PLHVs in wild boars is scarce [33,34]. The viruses classified in the subfamily *Gammaherpesvirinae* have not yet been proven to produce a clinical disease in pigs under natural conditions. However, experimentally immunocompromised miniature swine subjected to allogenic transplantation of hematopoietic stem cells developed clinical signs closely resembling those of *Human gammaherpesvirus 4* (Epstein-Barr virus; EBV)-associated postransplantation lymphoproliferative disease (PTLD) in immunosuppressed humans [35,36].

Regardless of significant advances in a methodology allowing thorough evaluation of epidemiological patterns of porcine herpesviruses, evidence for the molecular detection of beta- and gammaherpesviruses from a clinical specimen obtained from swine reared in Poland has not yet been published in peer-reviewed literature. Consequently, data on their phylogenetic relationships are not available. Therefore, the aim of this study was to demonstrate the possible infection, characterise the viruses belonging to *Betaherpesvirinae* and *Gammaherpesvirinae* and evaluate their potential role in contributing to coinfection with other important viral pathogens of swine based on clinical specimens obtained from pigs reared in a commercial, high-performing pig herd located in Poland.

## 2. Materials and Methods

### 2.1. Study Farm Characteristics

The present investigation was carried out at a 13,000-head weaner pig farm located in North-west Poland in January 2024. The pigs were born on a 3600-head sow farm, weaned after four weeks of the suckling period and transported to the location. All of the weaners were reared on slats in weekly batches in separate buildings following an all-in all-out system, under conditions meeting the legal welfare requirements of Council Directive 2008/120/EC of 18 December 2008, which lays down the minimum standards for the protection of pigs. The animals had unrestricted access to a wheat- and barley-based, dry-steam-conditioned pelleted feed manufactured according to the Danish nutrient standards.

Both farms implemented rigid biosecurity measures, including shower in/shower out for farm personnel and all visitors, air inlets fitted with fine mesh, a rodent control programme, own transportation services, fencing with disinfection gates, own feed mill and purchase of animals from one proven source only. The herds were toxigenic *Pasteurella multocida*-negative, porcine reproductive and respiratory syndrome virus (PRRSV)-negative, *Actinobacillus pleuropneumoniae* (APP)-negative, *Mycoplasma (M.) hyopneumoniae*-positive, influenza A virus (IAV-S)-positive as tested by IVD Innovative Veterinary Diagnostics (Seelze-Letter, Germany) using a combination of diagnostic testes (PCR, serology, microbiological culture) relevant to a particular pathogen. All animals received the intradermal vaccination against mycoplasmal pneumonia (Porcilis M Hyo ID ONCE; Intervet International B.V., Boxmeer, The Netherlands) and porcine circovirus 2 (PCV2)-associated diseases (Porcilis PCV ID; Intervet International B.V., Boxmeer, The Netherlands) at the age of five weeks. The animals were not immunised using vaccines against herpesviruses.

### 2.2. Samples

Nasal swabs were taken from 45 pigs exhibiting mild respiratory signs (sneezing and nasal discharge) following an age-dependent profile (15 individuals from each group) representing animals at 6, 8 and 10 weeks of age, i.e., 7 kg, 13 kg and 20 kg live weight, respectively. The research material was collected by a veterinarian with a sterile aluminium shaft and cotton tip swabs (DELTALAB S.L., Rubí Barcelona, Spain) by pushing the swab deep into the nose and rotating, ensuring not to contaminate the swab with surface microorganisms. Each sample was placed in an individual container with viral transportation medium (350C Copan Universal Transport Medium System; COPAN Diagnostics Inc., Murrieta, CA, USA), labelled, shipped overnight in a polystyrene box to the National Veterinary Research Institute (Puławy, Poland) and processed on the following day employing methodology described hereinafter. Additionally, samples of full blood collected from wild boar sampled during the 2013 hunting season from Opolskie, Pomerania, and Western Pomerania provinces, which were previously identified as gammaherpesvirus-positive by the VanDevanter et al. method [37], were used as a reference.

### 2.3. Detection of Herpesviruses and Other Viruses in Clinical Samples

DNA was extracted from 200 µL of samples using an IndiMag Pathogen Kit w/o plastics (INDICAL Bioscience GmbH, Leipzig, Germany) according to the manufacturer’s guidelines. The genetic material was eluted in 90 µL of elution buffer and stored at −65 °C until testing. Nested PCR designed for alpha-, beta- and gammaherpesvirus (pan-herpesvirus) DNA polymerase (DPol) gene detection was performed using primers described by VanDevanter et al. [37]. The first reaction was run in a 28 µL reaction mix that comprised 10 µL of water, 13 µL of ALLin HS Red Taq Mastermix 2x (highQu GmbH, Kraichtal, Germany), 1 µL of each of the two forward primers DFA: 5′ GAY TTY GCN AGY YTN TAY CC3′ and ILK: 5′TCC TGG ACA AGC AGC ARN YSG CNM TNA A3′ and reverse primer KG1: 5′ GTC TTG CTC ACC AGN TCN ACN CCY TT3′ (20 µM) and 2 µL of DNA sample. After 5 min of incubation at 94 °C, 45 cycles of amplification were run, each consisting of 30 s of denaturation at 94 °C, 60 s of annealing at 46 °C and 60 s of elongation at 72 °C. The reaction was finished with 7 min incubation at 72 °C. The second reaction was run using the same reaction mix and another set of primers, i.e., forward primer TGV: 5′TGT AAC TCG GTG TAY GGN TTY ACN GGN GT3′ and reverse primer IYG: 5′CAC AGA GTC CGT RTC NCC RTA DAT3′. After 5 min of incubation at 94 °C, 45 cycles of amplification were run, each consisting of 30 s of denaturation at 94 °C, 60 s of annealing at 46 °C and 60 s of elongation at 72 °C. The reaction was finished with 7 min incubation at 72 °C. Then, 6 μL of each PCR product was visualised on 3% agarose. Positive samples were expected to produce a 215–315 bp band.

All samples were also tested for the presence of influenza A virus (IAV), porcine parainfluenza Virus 1 (PPIV-1) and porcine reproductive and respiratory syndrome virus (PRRSV). The presence of IAV and PRRSV genetic material was detected using commercial qRT-PCR tests, the virotype influenza A 2.0 RT-PCR kit (INDICAL BIOSCIENCE GmbH, Leipzig, Germany) and the virotype PRRSV RT-PCR kit (INDICAL BIOSCIENCE GmbH, Leipzig, Germany). Samples positive for IAV (Ct < 37) were further tested for pandemic IAV H1 subtype using the Kylt^®^ Influenza A-H1 pdm Real-Time RT-PCR test (SAN Group Biotech Germany GmbH, Höltinghausen, Germany). All commercial assays were carried out according to the manufacturer’s protocols. Detection of PPIV-1 was conducted using an in-house qRT-PCR targeting the virus’s ORF2 gene [38].

### 2.4. Sequencing and Phylogenetic Analysis

Nested PCR products were sequenced using the Sanger method with previously described primers TGV and IYG. Sequencing was performed by the Genomed S.A. (Warsaw, Poland) company. The resulting partial sequences of the herpesvirus DPol gene were aligned, using MEGA-11.0.13. software, with sequences of previously identified herpesviruses originating from suids and other animals, available in GenBank. Alignment was used to construct a neighbour-joining phylogenetic tree [39]. Tree topology was evaluated with 1000 bootstrap replicates. Sequence identity estimation was performed using MEGA-11.0.13. software, and an identity matrix was constructed for isolated gammaherpesviruses and betaherpesviruses separately in relation to reference strains.

### 2.5. Statistical Analysis

The chi-squared (*χ*^2^) test was used to estimate the association between co-infections with respiratory viruses, with *p* value ≤ 0.05 considered significant.

## 3. Results

### 3.1. PCR Reaction and Sequencing

Among 45 tested animals, 28 (62.2%) were found to be positive for the panherpesvirus-specific PCR reaction (Table 1). Only 3 out of 15 (20.0%) animals were positive in the youngest age group (6-week-old pigs), while in both older groups (8 and 10 weeks) this rate was 12/15 (80.0%). Sanger sequencing of PCR-positive samples revealed the presence of animals infected with both SuHV-2/PCMV and SuHV-3/PLHV-1 in each of the age groups tested, but no other herpesviruses were identified. Partial sequences of DNA polymerase of identified herpesviruses were submitted to NCBI GenBank under accession numbers PP471446—PP471456 (PLHV-1 isolates) and PP471457—PP471469 (PCMV isolates).

### 3.2. Phylogenetic Analysis

**Figure 2:**
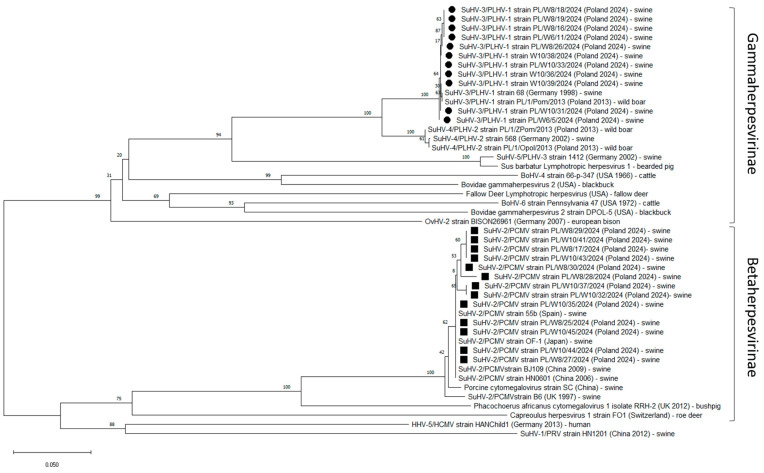
Neighbour-joining phylogenetic tree (1000 bootstrap replicates) of herpesvirus sequences obtained in this study. The percentage of replicate trees in which the associated taxa clustered together in the bootstrap test (1000 replicates) is shown above the branches The tree was constructed based on 149-nucleotide-long partial sequences of the DNA polymerase gene. Sequences of gammaherpesviruses acquired in this study are marked by black circles while those of betaherpesviruses are marked with black squares. The country, date of isolation (when available) and host species are included next to the name of the viral strain. Scale in the footer shows p-distance between branches.

In total, among 13 PCMV sequences analysed, 97.9–100% identity in the analysed fragment of DNA polymerase was observed. High identity (97.3–100%) was also observed with previously identified PCMV strains from China (B6—AJ222640, BJ109—KF017583, SC—HQ113116, HN0601—HQ686081), Spain (55b—AF268040), Japan (OF-1—AF268041), Germany (489—AF268042) and the United Kingdom (B6—AJ222640) [7,39] (Figure 2, Table S1).

All 11 PLHV-1 isolates identified in this study had identical nucleotide sequences in the analysed fragment of the DPol gene. Additionally, a high degree of identity (99.2–100%) was found with previously identified PLHV-1 strains isolated from swine in Germany in 1998 (PLHV-1 strain 68—AF118399) [22] and wild boar in Poland in 2013 (PLHV-1/PL/Pom/1/2013—PP444875) (this study) (Figure 1, Table S2).

### 3.3. Statistical Analysis of Rates of Coinfection

Regarding the detection rates of two herpesviruses evaluated statistically in our investigation (PCMV and PLHV-1), neither one was found to be significantly (*p* < 0.05) associated with the detection rates of IAV and PPIV1 (Table 2).

## 4. Discussion

Since their discovery, the biological features of porcine viruses belonging to the subfamilies *Beta*- and *Gammaherpesvirinae* have been insufficiently investigated. Overall, very few papers published to date allow authors to draw valid conclusions regarding their clinical relevance and potential synergistic pathogenicity in domestic pigs, including their contribution to the development of swine respiratory diseases. Nevertheless, molecular detection of novel agents in specimens collected from clinically affected swine populations that tested negative for the most common primary pathogens strongly emphasises the need for careful reconsideration of differential diagnosis procedures.

The investigation carried out on nasal swabs collected from clinically affected pigs reared on a commercial farm in Poland confirmed the presence of PCMV and PLHV-1. To the best of our knowledge, this study is the very first one describing the successful detection of the viruses in the local swine population. During this investigation, 13 PCMV and 11 PLHV-1 sequences were identified and formally submitted to the GenBank database. In general, in addition to their wide geographical distribution and genetic stability, the analysis revealed a high percentage of identity between the PLHV-1 sequences and the strain obtained from the wild boar population in Poland, highlighting the role of the latter as a potential carrier and reservoir of the pathogen in question.

Among all animals sampled during the investigation, the genetic material of PCMV and PLHV-1 was detected in 35.5% and 24.4% of weaners, respectively. Available scientific literature indicates that PCMV is a common finding in nasal swabs collected from weaned pigs. In Spain, PCMV belonged to the most frequently detected viruses among pigs from nurseries affected by outbreaks of respiratory diseases, accounting for 72.7% and 48.3% farm- and pig-level prevalence, respectively [10]. Similarly, our data regarding PLHV-1 prevalence are generally comparable with those obtained in Italy where 29% of animals reared in different locations (25% in healthy pigs, 33% in those exhibiting respiratory signs) provided PLHV-1-positive samples, i.e., blood, lymph nodes, lungs or nasal swabs [29]. Significantly higher PLHV-1 prevalence was described in pigs reared in Germany, where the distribution of PCR-positive blood, spleen and lung samples collected from commercial farms was 54%, 59% and 78%, respectively [23], and in the Republic of Ireland, with 74% positive spleen samples obtained from various abattoirs [27]. However, it must be noted that observed differences could also result from different diagnostic methods, as more sensitive qPCR was used in the studies mentioned above.

Our study was based on direct sequencing, which makes simultaneous identification of both pathogens in the case of mixed infection difficult. Hence, we cannot entirely exclude coinfections with both PLHV-1 and PCMV in individual pigs as was described before [40]. Nevertheless, as we tested for the presence of herpesviruses in the nasal swabs only, the overall prevalence of latent infections could be considered even higher. The shedding of the cell-free herpesvirus occurs usually in the youngest piglets, which then become latently infected, while the virus is associated with lymphatic cells as shown for PCMV [41]. Therefore, studies based on other biological matrices, i.e., spleen and lymph node testing, may present a significantly higher frequency of infections.

In our study, all of the tested animals showed mild respiratory signs; however, 40% of them were not positive for PLHV-1 or PCMV, and the investigation indicated a relatively low prevalence of other known viruses (11.1% IAV-positive individuals) or those suspected to produce such clinical signs (8.9% PPIV1 positive); these health issues might have been attributed to complex non-infectious aetiologies. Additionally, relationships between the detection rates of the herpesviruses and IAV/PPIV1 were not statistically significant. Stated differently, neither PLHV-1 nor PCMV infection described in the investigation contributed to viral coinfections. Nevertheless, previous studies showed that PCMV can not only cause respiratory diseases in infected animals but can also contribute to secondary bacterial or viral infections or parasitic invasions [10,42]. Although infections with PLHV were frequently found in swine herds, no clear association was proved between the presence of PLHV (alone or as a coinfecting agent) and clinical signs in those animals [29]. However, both PLHV and PCMV should also be considered as potential zoonotic agents. Previous studies showed that several viruses that have limited clinical importance when infecting swine or wild suids may have zoonotic potential, such as the hepatitis E virus, which can be transmitted to humans via contaminated food products [43,44]. While cytomegaloviruses were previously regarded as species-specific, it was observed that PCMV can also replicate in human cells in vitro [45]. Additionally, in the case of both PLHV-1 and PCMV, there are concerns related to the possible transmission of these viruses from infected tissues to organ recipients in xenotransplantation [18,46].

The lowest percentage of PCMV- and PLHV-1-positive samples was found in the youngest groups of animals, i.e., 6.7% and 13.3%, respectively. This finding is in accordance with previous observations from Brazilian and German studies demonstrating an increase in the proportion of PLHV-1 positives with the age of the tested animals, with the youngest groups remaining largely unaffected by this gammaherpesvirus infection [28,47]. Similarly, it was previously described that the number of PCMV-positive swine is the largest in animals around 8–9 weeks of age when more than 50% of all individuals could become shedders of the virus [9,48]. This pattern was also observed in our study, with 53.3% of the 8-week-old pigs shedding PCMV, which could have been attributed to the role of maternal immunity effectively limiting the spread of the virus in the early postweaning period. Even though the issue of maternal immune protection against the infections in question has already been raised [47,49], its clinical significance remains largely unknown.

Similarly to porcine herpesviruses, the role of maternal immunity in PRV1 infection remains to be elucidated. Our investigation demonstrated that PRV1 RNA could only be detected in 8.9% of the 6-week-old animals. Even though the virus has already been proven to be highly prevalent in Poland and other European countries [50,51,52], data on its circulation patterns and clinical significance are extremely limited. The detection rates described in our study are similar to those found by Woźniak et al. for Polish commercial sow farms that breed replacement gilts internally [50], where relatively small groups of animals and their rearing under high internal biosecurity standards seem to collectively limit the PRV1 infection to ≤7-week-old animals. Nevertheless, the exact mechanisms underlying the observation merit further investigation.

Contrary to the above-described patterns recognised for PLHV-1 and PCMV, the genetic material of IAV was not detectable in any of the nasal swabs obtained from the youngest group of animals. The prevalence of the IA virus in the 8-week-old group (26.7%) was identical to the one noted for PLHV-1 but remained markedly lower than the proportion of PCMV-positive pigs (53.3%) of the same age. In opposition to the epidemiological picture noticed for the herpesviruses, the considerable decline of IAV-shedding animals in the batch representing 10-week-old pigs (6.7%) was sharply distinguished. The observation seems to have been closely associated with a relatively short incubation period of IAV and sudden onset of the disease followed by high morbidity and rapid recovery.

Nevertheless, it should be taken into consideration that the reasoning applied to the current epidemiological investigation can be greatly affected by a relatively low number of samples collected from one location only. Since the observation could have been heavily influenced by some peculiarities, further comparative studies involving a significantly greater number of swine herds and individuals, as well as the in-depth analysis of on-farm-specific factors, are required to reevaluate the epidemiological and clinical features of the herpesviruses being discussed.

## Figures and Tables

**Table 1:** Panherpesvirus nested PCR-positive animals in different age groups.

Age Group	Herpesvirus			SuHV-2/PCMV			SuHV-3/PLHV-1			IAV			PPIV1/PRV1		
Weeks of Age	*n*/*N*	%	*CI*	*n*/*N*	%	*CI*	*n*/*N*	%	*CI*	*n*/*N*	%	*CI*	*n*/*N*	%	*CI*
6	3/15	20.0	0.0–40.2	1/15	6.7	0.0–19.3	2/15	13.3	0.0–30.5	0/15	0.0	0.0	4/15	26.7	4.3–49.1
8	12/15	80.0	59.8–100	8/15	53.3	28.1–78.6	4/15	26.7	4.3–49.1	4/15	26.7	4.3–49.1	0/15	0.0	0.0
10	12/15	80.0	59.8–100	7/15	46.7	21.4–71.9	5/15	33.3	9.5–57.2	1/15	6.7	0.0–19.3	0/15	0.0	0.0
TOTAL	27/45	60.0	45.7–74.3	16/45	35.6	21.6–49.5	11/45	24.4	11.9–37.0	5/45	11.1	1.9–20.3	4/45	8.9	0.6–17.2

*n*—number of positive animals; *N*—all animals tested in the category; *CI*—confidence interval (95%); SuHV-2/PCMV—suid herpesvirus 2/porcine cytomegalovirus; SuHV-3/PLHV-1—suid herpesvirus 3/porcine lymphotropic herpesvirus 1; PRRSV—porcine reproductive and respiratory syndrome virus; IAV—influenza A virus; PPIV1/PRV1—porcine parainfluenza virus-1/porcine respirovirus 1. All tested individuals were PRRSV-negative, while 5/45 (11.1%) were found to be IAV-positive. IAV RNA was detected in 4/15 (26.7%) and 1/15 (6.7%) samples, representing 6- and 8-week-old weaners, respectively. No samples were IAV H1-pdm-positive. PPIV1 RNA was detected in 4/15 (26.7%) animals from the youngest group. Samples collected from all other pigs tested negative for PPIV1 (Table 1). Simultaneous detection of genetic material representing two different viruses was infrequent and found in 4/45 (8.9%) clinical samples, representing groups of 8- and 10-week-old pigs only. Coinfections of SuHV-2/PCMV with IAV and SuHV-3/PLHV-1 with IAV were detected in 3/45 (6.7%) and 1/45 (2.2%) sampled individuals, respectively.

**Table 2:** Descriptive statistics of the rates of coinfection.

Variable A	Variable B	*n*	n_A-neg_/n_B-neg_	n_A-pos_/n_B-neg_	n_B-pos_/n_a-neg_	n_B-pos_/n_a-pos_	χ^2^	*p*
SuHV-2/PCMV	IAV	45	27	13	2	3	1.4669	0.226
SuHV-2/PCMV	PPIV1	45	23	16	6	0	3.8196	0.051
SuHV-3/PLHV-1	IAV	45	30	10	4	1	0.0602	0.806
SuHV-3/PLHV-1	PPIV1/PRV1	45	28	11	6	0	2.2398	0.143

SuHV-2/PCMV—*Suid herpesvirus 2*/porcine cytomegalovirus; SuHV-3/PLHV-1—*Suid herpesvirus* 3/porcine lymphotropic herpesvirus 1; IAV—influenza A virus; PPIV1/PRV1—porcine parainfluenza virus-1/porcine respirovirus 1.

## Data Availability

The data used to support the findings of this study are included within the article.

## Supplementary Materials

The following supporting information can be downloaded at: https://www.mdpi.com/article/10.3390/pathogens14040396/s1. Table S1. Identity matrix of betaherpesvirus isolated from swines in the study (numbers 1–13) in relation to selected previously sequenced viruses of this subfamily (numbers 14–22). Table S2. Identity matrix of gammaherpesvirus isolated from swines in the study (numbers 1–11) in relation to selected previously sequenced viruses of this subfamily (numbers 12–24).
