# Supplementary material for: First Molecular Detection of Porcine Cytomegalovirus (PCMV) and Porcine Lymphotropic Herpesvirus (PLHV) in Domestic Pigs in Poland
Source: Pathogens. 2025 Apr 18;14(4):396. doi: 10.3390/pathogens14040396 (PMC12030665; doi:10.3390/pathogens14040396)
Supplement: Supplementary file 1 [file pathogens-14-00396-s001.zip › pathogens-3585987-supplementary.pdf]

Table S1. Identity matrix of betaherpesvirus isolated from swines in the study (numbers 1-13) in relation to selected previously sequenced viruses of this subfamily (numbers 14-22).

		1.	2.	3.	4.	5.	6.	7.	8.	9.	10.	11	12.	13.	14.	15.	16.	17.	18.	19.	20.	21.
**1.**	SuHV-2/PCMV strain PL/W10/35/2024 (Poland 2024) - swine																					
**2.**	SuHV-2/PCMV strain PL/W10/32/2024 (Poland 2024) -swine	99.32																				
**3.**	SuHV-2/PCMV strain PL/W10/37/2024 (Poland 2024) - swine	99.33	100.00																			
**4.**	SuHV-2/PCMV strain PL/W10/41/2024 (Poland 2024)- swine	99.31	99.31	98.62																		
**5.**	SuHV-2/PCMV strain PL/W10/43/2024 (Poland 2024) - swine	99.32	99.31	98.64	100.00																	
**6.**	SuHV-2/PCMV strain PL/W10/44/2024 (Poland 2024) - swine	100.00	99.32	99.33	99.31	99.32																
**7.**	SuHV-2/PCMV strain PL/W8/17/2024 (Poland 2024)- swine	99.32	99.31	98.64	100.00	100.00	99.32															
**8.**	SuHV-2/PCMV strain PL/W10/45/2024 (Poland 2024) - swine	100.00	99.32	99.33	99.31	99.32	100.00	99.32														
**9.**	SuHV-2/PCMV strain PL/W8/25/2024 (Poland 2024) - swine	100.00	99.32	99.32	99.31	99.32	100.00	99.32	100.00													
**10.**	SuHV-2/PCMV strain PL/W8/27/2024 (Poland 2024) - swine	100.00	99.32	99.33	99.31	99.32	100.00	99.32	100.00	100.00												
**11.**	SuHV-2/PCMV strain PL/W8/28/2024 (Poland 2024) - swine	98.65	97.95	97.97	98.62	98.63	98.65	98.63	98.65	98.64	98.65											
**12.**	SuHV-2/PCMV strain PL/W8/29/2024 (Poland 2024) - swine	99.32	99.31	98.63	100.00	100.00	99.32	100.00	99.32	99.32	99.32	98.63										
**13.**	SuHV-2/PCMV strain PL/W8/30/2024 (Poland 2024) - swine	100.00	99.31	99.32	100.00	100.00	100.00	100.00	100.00	100.00	100.00	99.31	100.00									
**14.**	SuHV-2/PCMV strain 55b (Spain) - swine	100.00	99.32	99.33	99.31	99.32	100.00	99.32	100.00	100.00	100.00	98.65	99.32	100.00								
**15.**	SuHV-2/PCMV strain OF-1 (Japan) - swine	100.00	99.32	99.33	99.31	99.32	100.00	99.32	100.00	100.00	100.00	98.65	99.32	100.00	100.00							
**16.**	SuHV-2/PCMVstrain B6 (UK 1997) - swine	97.99	97.26	97.32	97.24	97.28	97.99	97.28	97.99	97.97	97.99	96.62	97.26	97.95	97.99	97.99						
**17.**	SuHV-2/PCMVstrain BJ109 (China 2009) - swine	100.00	99.32	99.33	99.31	99.32	100.00	99.32	100.00	100.00	100.00	98.65	99.32	100.00	100.00	100.00	97.99					
**18.**	SuHV-2/PCMV strain HN0601 (China 2006) - swine	100.00	99.32	99.33	99.31	99.32	100.00	99.32	100.00	100.00	100.00	98.65	99.32	100.00	100.00	100.00	97.99	100.00				
**19.**	Porcine cytomegalovirus strain SC (China) - swine	98.66	97.95	97.99	97.93	97.96	98.66	97.96	98.66	98.65	98.66	97.30	97.95	98.63	98.66	98.66	97.99	98.66	98.66			
**20.**	Phacochoerus africanus cytomegalovirus 1 isolate RRH-2 (UK 2012) - bushpig	79.19	78.77	79.19	77.93	78.23	79.19	78.23	79.19	79.05	79.19	77.70	78.08	78.77	79.19	79.19	77.85	79.19	79.19	77.85		
**21.**	Capreoulus herpesvirus 1 strain FO1 (Switzerland) - roe deer	55.03	54.11	55.03	53.10	53.74	55.03	53.74	55.03	54.73	55.03	53.38	53.42	54.11	55.03	55.03	55.03	55.03	55.03	55.03	54.36	
**22.**	HHV-5/HCMV strain HANChild1 (Germany 2013) - human	52.41	51.05	51.72	51.41	51.39	52.41	51.39	52.41	52.41	52.41	50.69	51.05	51.75	52.41	52.41	53.10	52.41	52.41	51.72	51.72	44.83

Table S2. Identity matrix of gammaherpesvirus isolated from swines in the study (numbers 1-11) in relation to selected previously sequenced viruses of this subfamily (numbers 12-24).

		1.	2.	3.	4.	5.	6.	7.	8.	9.	10.	11.	12.	13.	14.	15.	16.	17.	18.	19.	20.	21.	22.	23.
**1.**	SuHV-3/PLHV-1 strain PL/W10/31/2024 (Poland 2024) - swine																							
**2.**	SuHV-3/PLHV-1 strain PL/W10/33/2024 (Poland 2024) - swine	100.00																						
**3.**	SuHV-3/PLHV-1 strain W10/36/2024 (Poland 2024) - swine	100.00	100.00																					
**4.**	SuHV-3/PLHV-1 strain W10/38/2024 (Poland 2024) - swine	100.00	100.00	100.00																				
**5.**	SuHV-3/PLHV-1 strain W10/39/2024 (Poland 2024) - swine	100.00	100.00	100.00	100.00																			
**6.**	SuHV-3/PLHV-1 strain PL/W6/11/2024 (Poland 2024) - swine	100.00	100.00	100.00	100.00	100.00																		
**7.**	SuHV-3/PLHV-1 strain PL/W6/5/2024 (Poland 2024) - swine	100.00	100.00	100.00	100.00	100.00	100.00																	
**8.**	SuHV-3/PLHV-1 strain PL/W8/16/2024 (Poland 2024) - swine	100.00	100.00	100.00	100.00	100.00	100.00	100.00																
**9.**	SuHV-3/PLHV-1 strain PL/W8/18/2024 (Poland 2024) - swine	100.00	100.00	100.00	100.00	100.00	100.00	100.00	100.00															
**10.**	SuHV-3/PLHV-1 strain PL/W8/19/2024 (Poland 2024) - swine	100.00	100.00	100.00	100.00	100.00	100.00	100.00	100.00	100.00														
**11.**	SuHV-3/PLHV-1 strain PL/W8/26/2024 (Poland 2024) - swine	100.00	100.00	100.00	100.00	100.00	100.00	100.00	100.00	100.00	100.00													
**12.**	SuHV-3/PLHV-1 strain 68 (Germany 1998) - swine	100.00	100.00	100.00	100.00	100.00	99.33	100.00	99.33	99.33	99.33	100.00												
**13.**	SuHV-3/PLHV-1 strain PL/1/Pom/2013 (Poland 2013) - wild boar	100.00	100.00	100.00	100.00	100.00	99.33	100.00	99.33	99.33	99.33	100.00	100.00											
**14.**	SuHV-4/PLHV-2 strain PL/1/Opol/2013 (Poland 2013) - wild boar	93.24	93.24	93.24	93.24	93.24	93.24	93.20	93.24	93.24	93.24	93.24	93.24	93.24										
**15.**	SuHV-4/PLHV-2 strain PL/1/ZPom/2013 (Poland 2013) - wild boar	93.88	93.88	93.88	93.88	93.88	93.88	93.84	93.88	93.88	93.88	93.88	93.88	93.88	100.00									
**16.**	SuHV-4/PLHV-2 strain 568 (Germany 2002) - swine	93.24	93.24	93.24	93.24	93.24	92.62	93.20	92.62	92.62	92.62	93.24	93.29	93.29	100.00	100.00								
**17.**	SuHV-5/PLHV-3 strain 1412 (Germany 2002) - swine	70.95	70.95	70.95	70.95	70.95	70.47	70.75	70.47	70.47	70.47	70.95	71.14	71.14	69.59	70.07	69.80							
**18.**	Sus barbatur Lymphotropic herpesvirus 1 - bearded pig	70.27	70.27	70.27	70.27	70.27	69.80	70.07	69.80	69.80	69.80	70.27	70.47	70.47	68.92	69.39	69.13	97.99						
**19.**	BoHV-4 strain 66-p-347 (USA 1966) - cattle	52.05	52.05	52.05	52.05	52.05	51.70	51.72	51.70	51.70	51.70	52.05	52.38	52.38	51.37	51.72	51.70	55.10	53.74					
**20.**	Bovidae gammaherpesvirus 2 (USA) - blackbuck	56.16	56.16	56.16	56.16	56.16	55.78	55.86	55.78	55.78	55.78	56.16	56.46	56.46	56.16	56.55	56.46	57.82	58.50	73.29				
**21.**	Fallow Deer Lymphotropic herpesvirus (USA) - fallow deer	53.38	53.38	53.38	53.38	53.38	53.02	53.06	53.02	53.02	53.02	53.38	53.69	53.69	54.05	53.74	54.36	57.72	57.05	52.38	54.42			
**22.**	BoHV-6 strain Pennsylvania 47 (USA 1972) - cattle	52.03	52.03	52.03	52.03	52.03	51.68	51.70	51.68	51.68	51.68	52.03	52.35	52.35	51.35	51.70	51.68	55.03	55.03	51.70	54.42	60.40		
**23.**	Bovidae gammaherpesvirus 2 strain DPOL-5 (USA) - blackbuck	56.08	56.08	56.08	56.08	56.08	55.70	55.78	55.70	55.70	55.70	56.08	56.38	56.38	54.73	55.10	55.03	57.05	57.05	56.46	54.42	61.07	69.13	
**24.**	OvHV-2 strain BISON26961 (Germany 2007) - european bison	57.64	57.64	57.64	57.64	57.64	57.24	57.34	57.24	57.24	57.24	57.64	57.93	57.93	59.03	58.74	59.31	60.00	59.31	55.56	52.78	55.86	57.24	55.86
